# CytoSeg 2.0: automated extraction of actin filaments

**DOI:** 10.1093/bioinformatics/btaa035

**Published:** 2020-01-23

**Authors:** Jacqueline Nowak, Kristin Gennermann, Staffan Persson, Zoran Nikoloski

**Affiliations:** b1 School of Biosciences, University of Melbourne, Parkville, VIC 3010, Australia; b2 Bioinformatics, Institute of Biochemistry and Biology, University of Potsdam, 14476 Potsdam, Germany; b3 Systems Biology and Mathematical Modeling, Max Planck Institute of Molecular Plant Physiology, 14476 Potsdam, Germany

## Abstract

**Motivation:**

Actin filaments (AFs) are dynamic structures that substantially change their organization over time. The dynamic behavior and the relatively low signal-to-noise ratio during live-cell imaging have rendered the quantification of the actin organization a difficult task.

**Results:**

We developed an automated image-based framework that extracts AFs from fluorescence microscopy images and represents them as networks, which are automatically analyzed to identify and compare biologically relevant features. Although the source code is freely available, we have now implemented the framework into a graphical user interface that can be installed as a Fiji plugin, thus enabling easy access by the research community.

**Availability and implementation:**

CytoSeg 2.0 is open-source software under the GPL and is available on Github: https://github.com/jnowak90/CytoSeg2.0.

**Supplementary information:**

Supplementary data are available at *Bioinformatics* online.

## 1 Introduction

The actin cytoskeleton underpins many cellular processes, such as cytoplasmic streaming, cell wall organization and trafficking of vesicles inside the cell (Derksen *et al.*, 1990). Together with microtubules, the actin filaments (AFs) provide the backbone of the cytoskeleton.

Plant AFs have been visualized via immunolabeling in fixed samples or through fluorescently tagged cytoskeleton-binding proteins, such as GFP-fABD2, Lifeact or mTalin (Kost *et al.*, 1998; Riedl *et al.*, 2008; Sheahan *et al.*, 2004; Wick *et al.*, 1981; Wilsen *et al.*, 2006). Measurements of the organization and behavior of the AFs have therefore been steadily improving (Yoneda *et al.*, 2007). Although automated frameworks for the analysis of microtubule organization and dynamics are well-established (Faulkner *et al.*, 2017; Kapoor *et al.*, 2019), it has proven more challenging to device-automated frameworks to quantify features of the actin cytoskeleton, mainly due to its rapid dynamics. Nevertheless, several automated frameworks for AF analyses are available, including measurements of length, orientation and intensity distribution of filaments (Alioscha-Perez *et al.*, 2016; Rogge *et al.*, 2017; Zhang *et al.*, 2017).

Recently, we published an automated framework which extracts networks from segmented AFs (Breuer *et al.*, 2017). We used transport-related network properties to quantify the organization of the actin cytoskeleton and showed that AFs in *Arabidopsis thaliana* hypocotyls are optimized for efficient transport. Moreover, our framework can be used to compare the actin cytoskeleton organization between different organisms and different cell types (Yu *et al.*, 2019) but can also be used for other types of biological systems (see Supplementary Material).

Yet, the framework was provided as plain code that needed manual adjustments for individual experiments. Therefore, we present a graphical user interface (GUI) called CytoSeg 2.0 that facilitates easy use of the published algorithms and individualized gauging of parameters. The GUI was developed as a plugin for Fiji, which is widely used image processing software for biologists (Schindelin *et al.*, 2012).

## 2 Implementation and functionality

The CytoSeg 2.0 GUI is built as a macro for the Fiji imaging software. The code can be downloaded from Github (https://github.com/jnowak90/CytoSeg2.0) and should be extracted in the plugins folder of the Fiji application, which makes the GUI visible in the Fiji plugins menu. To use the GUI, both Fiji and Python 3 have to be installed with related plugins and modules (listed on the Github page).

The GUI is built for the analysis of fluorescently tagged actin cytoskeleton image stacks from living cells in TIFF format. However, it is also possible to use the GUI for immunolabeled AFs or other types of filamentous structures (see Supplementary Material). The user can select a single image stack (e.g. image stack of different time points) or a folder of images/movies as input. Large image files should be used with caution due to long running times. Demo images of control and LatB-treated actin cytoskeletons are provided on the Github page.

**Fig. 1.**
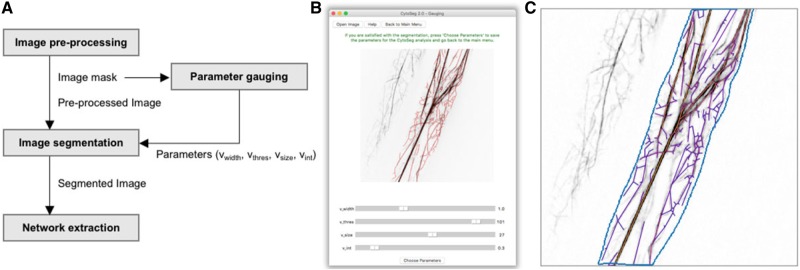
Overview of the CytoSeg 2.0 workflow. (**A**) Scheme of the four different steps of the image processing pipeline. (**B**) GUI for the parameter gauging and resulting segmented actin cytoskeleton (red). (**C**) Pre-processed image with the selected region of interest (blue) and overlayed extracted network (purple/orange)

The pipeline of CytoSeg 2.0 is partitioned into four steps: image pre-processing, parameter gauging, image segmentation and network extraction (Fig. 1A). The user can select whether to choose a complete analysis which includes all four steps, or a specific step of this series. During the pre-processing, the image is corrected for cell drift (stack registration), loss of fluorescence due to long light exposure (bleach correction) and uneven illuminated background (background subtraction). Maximum intensity Z-projected images are then used to manually select the region of interest, stored as an image mask (Fig. 1C). The mask is necessary for both the parameter gauging and the image segmentation. The segmentation of the image into actin cytoskeleton and background is dependent on four parameters: *v*_width_ (filament width), *v*_thres_ (adaptive median threshold), *v*_size_ (size of smallest components) and *v*_int_ (average filament intensity). To find the parameters for the optimal segmentation of the actin cytoskeleton, we added a GUI for the gauging of the parameters (Fig. 1B).

Here, the user can change the four parameters by dragging the corresponding sliders. The resulting segmented, and skeletonized actin cytoskeleton is then highlighted (red, Fig. 1B), and changes can be made until optimal segmentation is achieved. The selected parameters can be saved and will be stored for future analysis. To make sure that the selected parameters can be used for multiple images of the same experiment, several images should be tested during the gauging process.

Once the gauged parameters are selected, they can be used for the image segmentation. The segmented image is obtained by using a Gaussian filter (*v*_width_), adaptive thresholding (*v*_width_, *v*_thres_), removal of small particles (*v*_size_) and removal of filaments below a certain threshold (*v*_int_).

Networks are then extracted from the segmented image by defining crossings or endpoints of the skeleton as nodes, connected by edges if they can be directly reached on the skeleton. Furthermore, the edges are weighted according to their edge capacity which is defined by the weight and length of the underlying filament (Fig. 1C). Apart from the extracted networks, the algorithm also creates randomized networks that maintain the edge length distribution and number of nodes.

The resulting extracted and random networks, as well as a table of calculated transport-related network properties (average edge capacity, assortativity, number of connected components, average path length, algebraic connectivity and edge angles) are saved in a new folder for every image and can be used for further analysis, such as for comparison of network properties between different conditions, alteration of properties over time or testing for statistical significance.

## Supplementary Material

btaa035_Supplementary_Data
