# Supplementary material for: CytoSeg 2.0: automated extraction of actin filaments
Source: Bioinformatics. 2020 Jan 23;36(9):2950–1. doi: 10.1093/bioinformatics/btaa035 (PMC7203740; doi:10.1093/bioinformatics/btaa035)
Supplement: btaa035_Supplementary_Data [file btaa035_supplementary_data.docx]

**Supplementary Data**

We chose images of filamentous structures from different organisms to show the variety of problems to which CytoSeg 2.0 can be effectively used. First, we selected an image from the DRIVE database which shows the blood vessels in the retina (Staal *et al.,* 2004). Second, we selected an image of the dragonfly *Didymops transvera* by Phil Myers from the Animal Diversity Web (Myers *et al.,* 2019). Here, we used the wing pattern for segmentation. Last, we selected an image from the Cleared Leaves database which contains leaf images with enhanced veins. We selected a micro CT image of *Quercus faginea* (CLID_image_420_95918), provided by the Herbarium Senckenberianum (maintained by the department of Botany and Molecular Evolution, Senckenberg research Institute and Museum, Frankfurt/Main, Germany).

The images were individually processed using the same procedures as mentioned in the manuscript. We made some adjustments during the image pre-processing to ensure that the framework recognizes the selected features. The resulting extracted networks are shown in Figure S1.

**Fig. S1. Network extraction for different organisms.** A. Retina image from the DRIVE database, B. image of dragonfly wings from the Animal Diversity Web and C. leaf image from the Cleared Leaf Image Database. The corresponding, overlaid extracted networks are shown D.-F. The networks are colored according to their edge capacity (see color bar).

References

Das, A. *et al.* (2014) ClearedLeavesDB: an online database of cleared plant leaf images. *Plant Methods*, **10**, 8.

Myers, P. R. *et al.* (2019) The Animal Diversity Web (online). *Accessed at* [*https://animaldiversity.org*](https://animaldiversity.org) (19.08.2019).

Staal, J. J. *et al.* (2004) Ridge based vessel segmentation in color images of the retina. *IEEE Transactions on Medical Imaging,* **23**, 501-509.
